# A molecular approach to unravel trophic interactions between parasitoids and hyperparasitoids associated with pecan aphids

**DOI:** 10.1093/jisesa/ieae071

**Published:** 2024-07-11

**Authors:** Eddie K Slusher, Ted Cottrell, Tara Gariepy, Angelita Acebes-Doria, Marina Querejeta Coma, Pedro F S Toledo, Jason M Schmidt

**Affiliations:** Department of Entomology, University of Georgia, Tifton, GA, USA; USDA-ARS Southeastern Fruit and Tree Nut Research Laboratory, Byron, GA, USA; Texas A&M Agrilife Research and Extension Center, Stephenville, TX, USA; USDA-ARS Southeastern Fruit and Tree Nut Research Laboratory, Byron, GA, USA; Agriculture and Agri-Food Canada, London, ON, Canada; USDA-ARS Pacific Basin Agricultural Research Center, Hilo, Hawaii, USA; Institut de Recherche sur la Biologie de l’Insecte (IRBI), Université de Tours, Tours, France; Department of Functional Biology, University of Oviedo, Asturias, Spain; Department of Entomology, University of Georgia, Tifton, GA, USA; Department of Entomology, University of Georgia, Tifton, GA, USA

**Keywords:** aphid-parasitoid interactions, *Melanocallis caryaefoliae*, *Monelliopsis pecanis*, *Monellia caryella*, *Aphelinus perpallidus*

## Abstract

Advances in molecular ecology can overcome many challenges in understanding host–parasitoid interactions. Genetic characterization of the key-players in systems helps to confirm species and identify trophic linkages essential for ecological service delivery by biological control agents; however, relatively few agroecosystems have been explored using this approach. Pecan production consists of a large tree perennial system containing an assortment of seasonal pests and natural enemies. As a first step to characterizing host–parasitoid associations in pecan food webs, we focus on aphid species and their parasitoids. Based on DNA barcoding of field-collected and reared specimens, we confirmed the presence of 3 species of aphid, one family of primary parasitoids, and 5 species of hyperparasitoids. By applying metabarcoding to field-collected aphid mummies, we were able to identify multiple species within each aphid mummy to unravel a complex food web of 3 aphids, 2 primary parasitoids, and upward of 8 hyperparasitoid species. The results of this study demonstrate that multiple hyperparasitoid species attack a single primary parasitoid of pecan aphids, which may have negative consequences for successful aphid biological control. Although further research is needed on a broader spatial scale, our results suggest multiple species exist in this system and may suggest a complex set of interactions between parasitoids, hyperparasitoids, and the 3 aphid species. This was the first time that many of these species have been characterized and demonstrates the application of novel approaches to analyze the aphid-parasitoid food webs in pecans and other tree crop systems.

## Introduction

Elucidating trophic interactions between herbivorous insects and their parasitoids is important for estimating the impact of biocontrol services (Memmott et al. 1994, Hrcek et al. 2013), but data are still lacking for most host–parasitoid networks (Miller et al. 2021). This is in part due to challenges associated with rearing and identification of hosts and parasitoids (Memmott et al. 1994, Hrcek et al. 2013), the possibility of multiparasitoid interactions within the same host (e.g., multiparasitism and hyperparasitism), and the fact that traditional rearing, when successful, only reveals the ‘winner’ of these interactions (Gómez-Marco et al. 2015, Lefort et al. 2017). Furthermore, host rearing does not guarantee parasitoid emergence due to host and/or parasitoid mortality, thus leading to an underestimation of the diversity, abundance, and impact of parasitoid species present in a system (Gómez-Marco et al. 2015, Lefort et al. 2017, Šigut et al. 2017, Abram et al. 2019, Kitson et al. 2019, Sow et al. 2019).

Molecular analysis can facilitate identification of host–parasitoid–hyperparasitoid interactions (Moritz and Cicero 2004, Smith et al. 2008, Gariepy and Messing 2012, Taberlet et al. 2018); however, the lack of publicly available, reliable, and accurate sequence data can make it challenging to fully realize the complexity and value of host–parasitoid interactions and the ecosystem services they provide (Taberlet et al. 2018, Miller et al. 2021). Regardless, DNA-based tools can help unravel complex food–web interactions when multiple parasitoids and hyperparasitoids are known to attack a given host species. These tools have advanced our understanding of the biological control of crop pests such as aphids, where several studies have unraveled trophic interactions through analysis of mixed DNA samples (e.g., such as DNA contained in parasitized aphids, hereafter referred to as mummies) using meta-barcoding (Traugott et al. 2006, 2008, Heraty et al. 2007, Bon et al. 2008, Desneux et al. 2009, Verennes et al. 2014, Lefort et al. 2017, Gariepy et al. 2019, Sow et al. 2019). Many aphid species are economically important crop pests, and therefore, often have adequate sequence data in public archives to facilitate identification through molecular analysis (Traugott et al. 2008, Gariepy and Messing 2012, Zhou et al. 2014, Gómez-Marco et al. 2015, Lefort et al. 2017, Ye et al. 2017b, 2017a, Zhu et al. 2019). Further, the availability of algorithms that assign specimens to operational taxonomic units (OTUs) based on their DNA barcodes provides interim taxonomy (in the form of Barcode Index Numbers, BINs) to unidentified specimens, thereby allowing biodiversity assessments (Ratnasingham and Hebert 2013) The availability of a BIN system for assessing biodiversity in a given cropping system is particularly advantageous for aphids dwelling in orchard crops, and more specifically tree nuts such as pecans (*Carya illinoinensis*), which can be considered underrepresented aphid systems (Wood et al. 1987, Honaker et al. 2013).

Pecans, unlike other tree crops, host the season-long (and often concurrent) occurrence of 3 aphid species of economic concern: yellow pecan aphid, *Monelliopsis pecanis* Bissell, blackmargined aphid, *Monellia caryella* (Fitch), and black pecan aphid, *Melanocallis caryaefoliae* (Davis) (Hemiptera: Aphididae) (Tedders 1978, Cottrell et al. 2009, Paulsen et al. 2013). Within the pecan orchard system, at least one primary parasitoid species, *Aphelinus perpallidus* Gahan (Hymenoptera: Aphelinidae), is known to specialize on the pecan aphid complex where it parasitizes *M. pecanis*, *M. caryella*, and, to a much lesser extent, *M. caryaefoliae* (Bueno and Stone 1983, 1985). Previous research also recognizes the occurrence of other aphid parasitoid and hyperparasitoid species in the pecan system (Bueno and Stone 1983, Slusher et al. 2021), suggesting the existence of complex trophic links that remain unresolved by conventional rearing and observation.

Here, we used traditional DNA barcoding to generate initial molecular data to characterize the key players in the pecan aphid–parasitoid–hyperparasitoid food web, and applied DNA metabarcoding of aphid mummies to unravel potential trophic links in the system. We focused on characterizing the food web in Central and South Georgia, where pecan aphid can be a major pest. Prior to this study, sequence data for 2 of the pecan aphids, *M. pecanis* and *M. caryaefoliae*, were not represented in public databases (BOLD and GenBank), and knowledge of which hyperparasitoids occur in the pecan aphid system was unknown. Understanding the hyperparasitoid assemblage associated with *A. perpallidus* will help to determine the biological control potential of *A. perpallidus* in pecan orchards. In addition, due to the difference in age and preservation quality of our samples, we also evaluated and compared the quality of DNA extracted from individual mummies that were (1) freshly collected (<24 h from collection), (2) preserved at −20 °C for 1 yr, or (2) stored at room-temperature for 1 yr or greater. This study intends to lay the groundwork for future host-parasitoid network and network dynamic studies in relation to aphid management practices in pecan crops.

## Materials and Methods

### Sample Collection and Study System

#### Aphid Acquisition

All 3 aphid species recognized in pecan systems (Tedders 1978, Cottrell et al. 2009, Paulsen et al. 2013): *M. pecanis*, *M. caryella*, and *M. caryaefoliae* were lab-reared on open-pollinated Sumner variety pecan seedlings in Tift County, Georgia, USA. Twenty adult aphids of each species were isolated and placed in 70% ethanol for preservation, then frozen at −20 °C. Species identity was confirmed based on taxonomic descriptions by Tedders (1978). Twenty of each aphid species were prepared for DNA barcoding (Sanger sequencing).

#### Parasitoid Acquisition and Comparison of Sample Age for Mummies

All sampling sites were commercial orchards in major pecan growing regions in central and southern Georgia, USA. All sites were treated with insecticides for aphid management; however, insecticide applications varied according to site ranging from one insecticide application a year (Dougherty and Macon) to up to 8 insecticide applications per year. At each location, 10 mature ‘Sumner’ pecan trees were randomly selected. Using a pole pruner, 5 compound leaves were collected from the lower canopy (approximately 1–2 m from the ground) of each tree. To standardize the sampling, only the 3 middle pairs of leaflets from each leaf were collected and examined. Each leaf was stored in a labeled 3.79 L Ziploc bag. Leaf samples were stored in a cooler with ice blocks during transit and were immediately transferred to a refrigerator upon arrival in the lab. Within 48 h of collection, mummies were examined to determine whether parasitoids had emerged prior to leaf collection. Mummies from which parasitoids had not emerged were gently removed from the leaf and were placed individually in plastic capsules (size 0, 7.62 cm, Healthy Life Supply; Mound House, NV). Parasitoids that emerged from these mummies were identified as either primary or hyperparasitoids using a reference collection identified by a parasitic hymenopteran taxonomist, James Woolley (Texas A&M University), and morphological descriptions by Goulet and Huber (1993). Emerged parasitoids and hyperparasitoids were stored in ethanol following emergence and were used for subsequent DNA barcoding. Any remaining unemerged mummies (*n* = 248) were used for metabarcoding in a balanced design to explore sample age and preservation history on detection of parasitoid food webs. To examine the effects of age and sample preservation, we prepared unemerged mummies in 3 categories:

(1) Fresh mummies collected in 2021 from a commercial pecan orchard in Macon County, Georgia, USA (32°30ʹ00.8″N, 83°55ʹ46.8″W) that were preserved in 70% ethanol and stored at −20 °C immediately following collection, with DNA extracted within 1-day post collection (*n* = 84).
(2) Frozen mummies collected in 2020 from commercial pecan orchards in Lowndes County, Georgia, USA (31°01ʹ18.2″N 83°14ʹ40.8″W) and Macon County, Georgia, USA and placed in 70% ethanol and stored at −20 °C for 1 yr prior to DNA extraction (*n* = 82).
(3) Mummies stored at 25 °C for ≥1 yr following collection (*n* = 82). These specimens were collected between 2018 and 2020 from commercial pecan orchards in Brooks County (2018) (31°0ʹ26.40″N, 83°28ʹ58.72″W), Berrien County (2020) (31°04ʹ01.3″N 83°11ʹ08.8″W), Dougherty County (2019) (31°36ʹ12.1″N, 84°02ʹ34.0″W), Lowndes County (2019), and Macon County (2018, 2019, 2020) (All Georgia, USA). Specimens were placed individually in plastic capsules and stored in an environmental chamber (25 °C, 60% RH, 16:8 L:D, Percival E36L2; Perry, IA) for at least 30 days to check for parasitoid emergence. Unemerged mummies were stored in 70% ethanol at room temperature for ≥1 yr prior to DNA extraction.

### DNA Extraction, Sequencing, and Bioinformatics

#### DNA Extraction

Genomic DNA was extracted from colony aphids, emerged parasitoids, and aphid mummies using Qiagen DNeasy Blood and Tissue 96-well kits (including a negative control containing all buffers and solutions, but no insect tissue) following the manufacturer’s protocol (Qiagen, Chatsworth, CA, USA). Extracted DNA was stored at −20 °C. Aphids from laboratory colonies and emerged parasitoids were used for Sanger sequencing, and all aphid mummies (unemerged) were sequenced using metabarcoding on the Illumina MiSeq platform.

#### DNA Barcoding of Aphids and Emerged Parasitoids

DNA extractions were amplified using published primers following standard protocols (Folmer et al. 1994). PCR reactions (10 μl) contained 5 μl/rxn 2× Qiagen multiplex master mix, 0.1 μl/rxn BSA, 1.9 μl/rxn PCR grade H_2_O, and 0.5 μl/rxn of either LCO1490 or HCO2198 primers. PCR reactions were run using a Bio-Rad C1000 Touch Thermal Cycler. PCR protocol was performed as follows: 95 °C for 15 min, 94 °C for 30 s, 53.3 °C for 40 s, 73° C for 1 min, 94 °C for 30 s, 46.6 °C for 1 min, 73 °C for 1:30 min, and a final extension of 72 °C for 10 min. The PCR products were bi-directionally sequenced on an ABI 3730 DNA Analyzer (Eurofins Genomics LLC). Following sequencing, forward and reverse sequences were assembled, aligned, and edited using Codon Code Aligner program, version 4.0.4. Resulting sequences were screened against all barcode records in the Barcode of Life Datasystems (BOLD) and NCBI to identify the samples to the lowest possible taxonomic groups.

#### DNA Metabarcoding of Aphid Mummies and Metabarcoding Bioinformatics

We used a 2-step nested DNA metabarcoding approach to estimate species of parasitoids and hyperparasitoids associated with aphid mummies (Kitson et al. 2019, Grabarczyk et al. 2022). DNA from 3 aphid mummy categories described above were isolated separately forming one sample of a mummy and used for PCR. In the first PCR, aphid mummy DNA was amplified with the mlep/lep (Hajibabaei et al. 2006, Lefort et al. 2017). The primers mlep/lep appear to be biased toward Hymenoptera (Lefort et al. 2017), when aphids mummies are the hosts, making them a good choice for attempting to reveal host–parasitoid–hyperparasitoid interactions. The primers contained Illumina bridging primers and dual tags of unique combinations of 8 forward and 8 reverse tags for uniquely labeling each aphid mummy sample (Kitson et al. 2019). PCR products were then cleaned with magnetic beads (MagBio HighPrep PCR, MagBio Genomics, Inc.) and clean PCR products were used as the template for the second PCR with primers for Illumina adaptors and dual-indexed to label each plate of samples. PCR products were cleaned, and concentration of amplicons standardized then pooled to form our library. Pooled samples were submitted to the Georgia Genomics and Bioinformatics Core lab (GGBC-UGA) for sequencing on an Illumina MiSeq (Illumina, San Diego, CA, USA) with V3 chemistry and 600 cycles.

Bioinformatic processing post-sequencing began with assessing library quality on both forward and reverse reads using Fast (Andrews 2010). Libraries were then DE multiplexed and primers on forward and reverse reads were trimmed using *cutadapt* software (Martin 2011). Following trimming, reads were merged with *PEAR* software (Zhang et al. 2014) applying a Phred score threshold of 30. Remaining filtering steps were performed using *VSEARCH v2.8.2* software (Rognes et al. 2016). Reads were filtered by quality (*fastq_maxee* = 1) and dereplicated (Gordon and Hannon 2010). Subsequently, singletons, indels, and chimera were removed from the sequence dataset until obtaining amplicon single variants (ASVs). Finally, ASVs were clustered applying a cut-off threshold of 97% into OTUs, which were listed in a *fasta* file. Final mapping was performed to obtain the by-sample OTU table. Taxonomic assignment of the sequences was done against NCBI GenBank nr/nt BLAST algorithm and R package tazonomizr (https://github.com/sherrillmix/taxonomizr/)(Johnson et al. 2008). OTU tables were based on reads of taxa recovered from mummies that contained Hymenoptera and Hemiptera DNA, and included only the clusters that could be identified to family level with >95% identity, and >300 bp length for mlep. We then removed rare reads with a threshold for detection of any taxa set at >10 reads, and any taxon with <10 reads was adjusted to zero (i.e., absent; Sullins et al. 2018, McClenaghan et al. 2019, Stolz 2019).

### Statistical Analysis

We estimated descriptive statistics and conducted ANOVA to compare mummy age effects on recovering parasitoid reads in R version 4.1.2 ‘Bird Hippie’ (RCoreTeam 2021). For analysis, we included only samples with >10 reads of either Hymenoptera or Hemiptera.

## Results

### DNA Barcoding of Pecan Aphid–Parasitoid–Hyperparasitoid System

In total, 161 samples of aphids and emerged parasitoids were extracted and analyzed, with DNA barcodes generated for 106 high-quality samples (66%). Specimen data, DNA sequences, and tracefiles are publicly available on the Barcode of Life Data Systems (https://v4.boldsystems.org/) under project JSPCN, Diversity of aphids and aphid parasitoid in pecan orchards. All sequences are also available in Genbank (OK345403–OK345508). The BioProject accession number for this project is PRJNA1119291. The samples included 60 pecan aphid specimens, 58 primary parasitoids reared from mummies, and 43 suspected hyperparasitoids reared from mummies. A total of 43 out of 60 aphid specimens were successfully amplified and sequenced, with DNA barcodes separating the specimens into 3 distinct species, consistent with the morphological identifications of lab-reared colony aphids: *M. caryaefoliae* (*N* = 14), *M. pecanis* (*N* = 13), and *M. caryella* (*N* = 16; Table 1). For emerged primary parasitoids, DNA from 47 out of 58 specimens were successfully amplified and sequenced (72%). Based on the COI sequences generated from the primary parasitoids, all but one were categorized with the same BIN suggesting the majority are a single species, *A. perpallidus*. The remaining BIN was identified as *Aphelinus* sp. (Table 1). Of the 43 emerged hyperparasitoid specimens extracted, only 16 successfully provided high quality DNA barcode sequences (37%). Based on sequence comparisons in Genbank and BOLD, we identified 2 species of Chalcidoidea (*Asaphes vulgaris*, *Pachyneuron* sp.), one unidentified species of Signiphoridae, and 2 species of Fitigidae (both in the genus *Alloxysta*; Table 1).

**Table 1.**
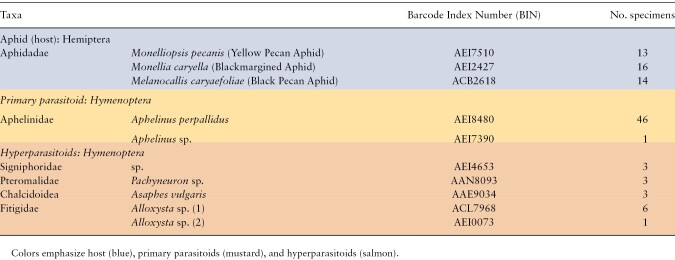
Summary of taxa and specimen identified at each trophic level using DNA Barcoding using LCO/HCO primers to amplify a 658-bp Folmer region of COI of whole body extractions of aphids and emerged parasitoids/hyperparasitoids

### Metabarcoding of Aphid Mummies

**Fig. 1.**
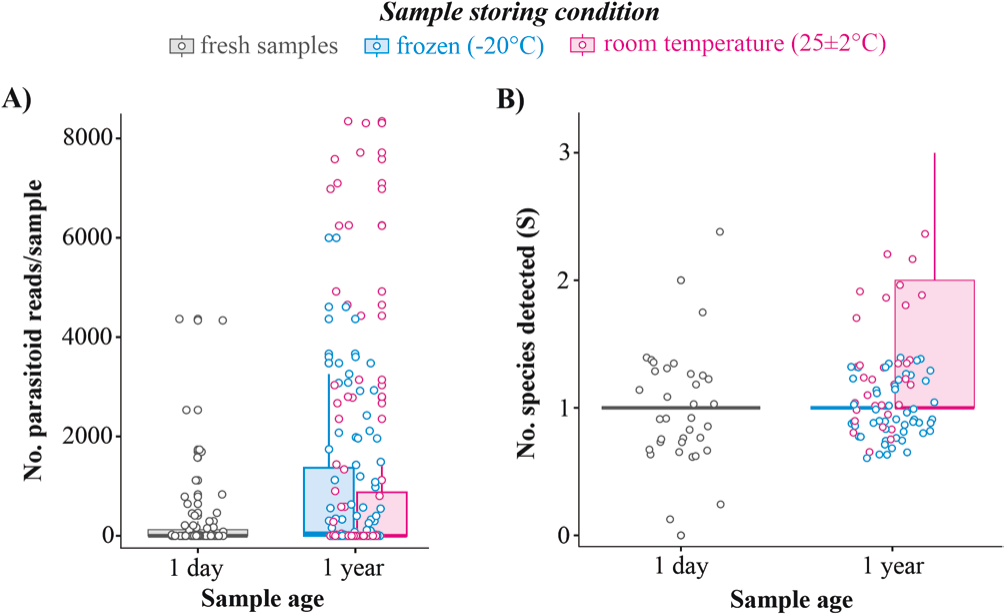
Comparison of parasitoid reads and richness of taxa recovered from aphid mummies.

**Fig. 2.**
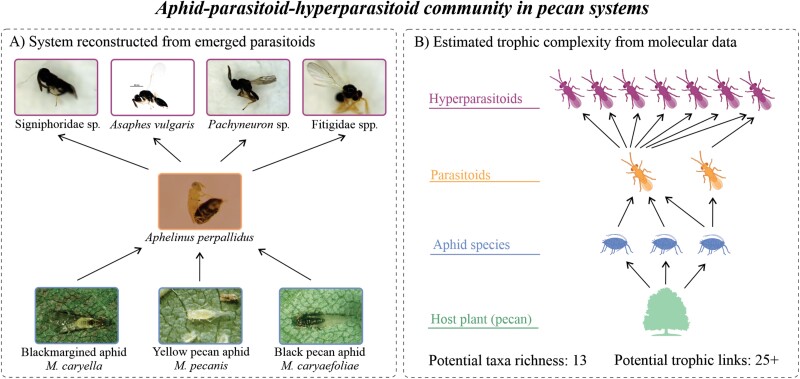
Current predicted aphid–parasitoid–hyperparasitoid food web based on (A) DNA barcoding of emerged parasitoids from field collected mummies, and (B) DNA barcoding combined with metabarcoding of aphid mummies for an estimated aphid–parasitoid–hyperparasitoid system in pecan orchards. For (B), richness and trophic link estimates are provided for combining taxa observed for this initial view of potential complexity in the aphid-pecan system. Pecan aphid photos are courtesy of Louis Tedders, USDA Agricultural Research Service, Bugwood.org. *Asaphes vulgaris* photo with permission from BOLDSYSTEMS (ASGLE-0322), Centre for Biodiversity Genomics.

We recovered a total of 2,238,286 raw reads of which 99% were successfully demultiplexed. Once trimmed and merged following quality filtering, we retained a total of 599,396 reads recovered with an average of 5828 reads per sample for Hymenoptera and Hemiptera for an average of 1,124 ± 1SD 2091 per sample. Reads for the 3 aphid species were recovered at very low levels (Table 2). The percentage of samples (i.e., 1 sample = 1 aphid mummy) with detectable levels (>10 reads for an OTU) of Hymenoptera DNA from aphid mummies was 52% (130/248). The majority of these reads were from primary parasitoids, including *A. perpallidus* 84% (109/130), and *Trioxys* sp. 1.6% (2/130). Reads were also recovered for taxa that are likely hyperparasitoids at 16% (21/130; Table 2), based on known literature reports of these families (Encyrtidae, Figitidae, Pteromalidae, Chalcidoidea, and Signiphoridae) acting as hyperparasitoids of aphids, rather than as primary parasitoids (Singh and Singh 2016). In addition, we detected both primary and hyperparasitoid DNA in 12 out of the 130 samples. Interestingly, despite having material left in the laboratory at 25 °C for an entire year prior to DNA handling and sequencing, we were able to recover reads from this material and significantly more reads for parasitoids and hyperparasitoids and more species per sample were recovered from older material (*F*_2,118_ = 5.44, *P* = 0.0054, *F*_2,118_ = 11.202, *P* *< *0.0001; Table 2, Fig. 1). Despite poor preservation conditions, these results provide evidence that metabarcoding can recover host–parasitoid species interactions for extended periods after collection. We hesitate to make any conclusions regarding the nature of the difference between these materials because the collections were not completed in a systematic way to remove confounding variables between years or balanced samples between sampling sites. Work was completed to have balanced replication between materials. Nonetheless, the combined data of DNA barcoding of emerged adult wasps and the metabarcoding of aphid mummies suggest a complex of species interactions containing 3 aphid species, 2 primary parasitoids and approximately 8 hyperparasitoids (Table 2, Fig. 2).

**Table 2.**
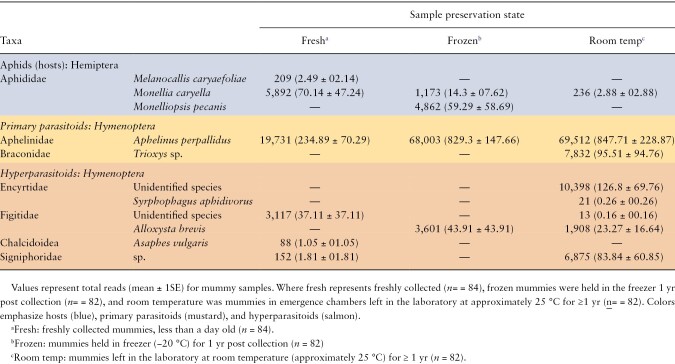
Summary of metabarcoding of aphid mummies using mlep/lep primers for a 350-bp target COI sequence within the Folmer region

## Discussion

This is the first study to provide a molecular characterization of the pecan aphid–parasitoid system. We generated new DNA barcode sequence data for 3 species of aphids, one species of primary parasitoid, and 5 species of hyperparasitoids (Table 1). The DNA sequence data confirm that aphids reared in our colonies established from field populations over the 3 yr of this study constitute 3 separate species. This corroborates the morphological assessment of the 3 aphids detailed by Tedders (1978). Although we uncovered 2 possible primary parasitoids, most of the recovered reads from mummies were from a single primary parasitoid species that appears to numerically dominate this system (Slusher et al. 2021, Toledo et al. 2024) and potentially interacts with a diversity of hyperparasitoids (Table 1). We also found evidence of black pecan aphid parasitism in our samples (Fig. 2), despite prior studies suggesting that parasitism of black pecan aphids by *A. perpallidus* is rarely observed (Tedders 1978). However, all 3 aphid species produce swollen and shiny black mummies (Tedders 1978), thus it is possible that parasitism of black pecan aphids may be underrepresented due to the difficulty of differentiating parasitized aphid species.

Based on the DNA barcoding results, metabarcoding of mummies and past sampling in this system (Slusher et al. 2021, Toledo et al. 2024), the primary parasitoid in pecan systems in Georgia is *A. perpallidus*, a member of the family Aphelinidae that parasitizes pecan aphids (Tedders 1978, Bueno and Stone 1985). James Wooley, a parasitoid specialist formerly of Texas A&M, identified specimens of primary pecan aphid parasitoids submitted by the authors and revealed most to be *A. perpallidus*. It was noted, however, that some specimens were lighter in color and were possibly a different species. Distinct populations consisting of unique phenotypes of *A. perpallidus* have been previously reported (Dickey and Medina 2011). However, it is difficult to assess if cryptic species or different strains are present in this system due to lack of sequences for Aphelinidae present in pecan. Although we did get a single specimen that was identified as *Aphelinus* sp. based on DNA barcoding (Table 1), more extensive DNA sequencing of specimens from across the geographic range of *A. perpallidus* may provide more insight regarding the diversity of this and other *Aphelinus* species in nut trees.

Although many aphid mummies contained *A. perpallidus*, as the primary parasitoid, we also revealed evidence of a second primary parasitoid, *Trioxys sp*. (Hymenoptera: Braconidae) (Table 2). Two *Trioxys* species (*T. pallidus* Haliday and *T. complanatus* Quilis) were previously imported and released in Byron, Georgia pecan orchards (Tedders 1977). However, contemporary follow-up surveys failed to uncover evidence of their establishment. It is possible that the *Trioxys* recovered from our samples are derived from these original releases. Although both *T. pallidus* and *T. complanatus* have sequences in GenBank, these sequences did not match with our sequences. Another unidentified *Trioxys* species that emerged from a yellow pecan aphid mummy was also previously reported (Tedders 1978). Thus, additional studies are needed to follow up on this, perhaps with more extensive surveys to collect braconid aphid parasitoids in the pecan system. If more specimens can be obtained, then a more thorough barcode inventory may help resolve the identity and potential role of *Trioxys* species in this foodweb. With the current dataset, it appears that *Trioxys* sp. are present but at low numbers in relation to *A. perpallidus* and associated hyperparasitoids.

Our study is the first to characterize hyperparasitoids associated with *A. perpallidus* in the southeastern USA; however, we were unable to provide species-level identifications for most hyperparasitoid specimens. Despite the inability to get species-level identification, what we recovered provides evidence of a diversity of hyperparasitoid presence, rather than primary parasitoids. Members of the families: Encyrtidae, Figitidae, Pteromalidae, Chalcidoidea, and Signiphoridae are all acknowledged as hyperparasitoids of aphids, rather than as primary parasitoids (Singh and Singh 2016). Identification of hyperparasitoids is challenging in many food webs given their small size, difficult-to-distinguish morphological characters, the lack of taxonomic expertise for some hyperparasitoid families, and the absence of DNA sequence data in public databases for expertly identified specimens (Poelman et al. 2022).

However, DNA barcoding does provide BINs that can serve as interim taxonomy for individual species until these species can be expertly identified based on morphological and genetic characters (Ratnasingham and Hebert 2013). In the present study, we were able to describe at least 5 different hyperparasitoid BINs (putative species) from 3 different families (Figitidae, Pteromalidae, and Signophoridae; Table 1). In the near future, further investigation of this system should provide more clarity on species-level identifications that will allow updated taxonomy to be assigned to each BIN. Bueno and Stone (1985) previously identified *Alloxysta schlingeri* (Andrews) (Hymenoptera: Figitidae), *Signophora* spp. (Hymenoptera: Signophoridae), and *Aphidencyrtus* spp. (Hymenoptera: Encyrtidae) as hyperparasitoids of *A. perpallidus* in Texas using morphological methods. Our barcoding of parasitoids identified some specimens as belonging to the Signophoridae, as well as members of the genus *Alloxysta*; however, we were unable to identify these to the species level due to a lack of hyperparasitoids in public DNA databases. It is possible that *A. schlingeri* could be one species that we recovered from A. *perpallidus* in the southeast; however, DNA barcoding of expertly identified voucher specimens of *A. schlingeri* would be needed to clarify this, as there are currently no sequences for this species in BOLD or Genbank. *Asaphes vulgaris* Walker (Hymenoptera: Chalcidoidea) was found in the present study but was not found in previous studies in Texas; however, this species is commonly associated with other *Aphelinus* species (Brodeur and McNeil 1994), so it is not unexpected. Although there may be more hyperparasitoids in this system, we currently uncovered a minimum of 5 successfully emerging from pecan aphid mummies.

Based on Bueno and Stone (1985) and our study, it is reasonable to suggest that a single primary parasitoid is experiencing potential top-down pressure from multiple hyperparasitoids in the pecan aphid–parasitoid–hyperparasitoid food web (Fig. 2). All 5 putative species identified in our system are generalist hyperparasitoids of aphid parasitoids in many agricultural and natural habitats (Specht 1969, Dean et al. 1981, Powell 1982, Ferrer-Suay et al. 2014, Zamora-Mejías and Hanson 2016, Martens 2018, Taberlet et al. 2018, Bandyan et al. 2021). Given that most hyperparasitoids have broad host ranges and parasitize primary parasitoid species on a variety of aphid species on different host plants (Poelman et al. 2022), additional studies in pecan and other overlapping habitats may uncover more diverse assemblages and may reveal broader connectivity across food webs that are linked through generalist hyperparasitoids. Much remains to be learned about hyperparasitoid activity in many systems, and while studies commonly analyze primary parasitoid–host interactions, trophic interactions between primary parasitoids and hyperparasitoids is largely under studied (Franck et al. 2017, Kitson et al. 2019, Sow et al. 2019). In addition, hyperparasitoid competition for a single primary parasitoid host can lead to shifts in the trophic position of a given hyperparasitoid species (Poelman et al. 2022). For example, Lefort et al. (2017) suggest the existence of a 5th tropic level of hyperparasitism in their aphid–parasitoid–hyperparasitoid system and, the species richness of hyperparasitoids observed here, suggests a complex set of interactions occurring in the pecan systems.

Our study developed a preliminary picture of the pecan aphid–parasitoid–hyperparasitoid food web in pecan systems and suggests further analysis to develop a more complete understanding of how the key players in this (and neighboring) system interact. As reviewed by Poelman et al. (2022), hyperparasitoids can reduce the population of parasitoids occupying the third trophic level, which can in-turn lead to herbivore outbreaks when they are released from the pressure exerted by primary parasitoids. Previous work by Slusher et al. (2021) found the highest hyperparasitoid rates at sites with the highest amount of *A. perpallidus* present, indicating a density dependent relationship. This is well known in other hyperparasitoid systems, where the abundance and species richness of hyperparasitoids follows the abundance of primary parasitoids (Poelman et al. 2022). Bueno and Stone (1983) did not observe a significant impact of hyperparasitism on *A. perpallidus* biological control. The true effect of hyperparasitoids on *A. perpallidus* remains to be examined, but currently only a small percentage of mummies contained both primary and hyperparasitoid DNA. Therefore, while there is a diversity of hyperparasitoids in the system, low levels of hyperparasitoids observed in mummies and high populations of *A. perpallidus* observed in the system (Slusher et al. 2021, Toledo et al. 2024), suggests that *A. perpallidus* is not experiencing strong top-down pressure from hyperparasitism. But more controlled future research of the species composition of the broader aphid–parasitoid–hyperparasitoid community in the pecan system and adjacent habitats will advance our understanding of the community structure and potential impact of primary parasitoids and hyperparasitoids on biological control services in this system.

## Data Availability

Upon publication all data will be released to public databases for sequence archiving.
